# 

**Published:** 1883-12

**Authors:** 


### NOTE TO SUBSCRIBERS.

Subscribers, who lack any of our issues for *past* year will be supplied on application. Subscribe promptly for coming year.

### INDEX

TO THE

### HOMŒOPATHIC PHYSICIAN.

### VOLUME III.

PAGE

Aconite ....................26, 27, 64
Actea rac .................... 25, 64
Addresses to Associated Bodies. P. P. Wells .................... 141
After Pains, Notes on .................... 25
Agaricus ....................64, 90
Agnus c. .................... 325
Aloe .................... 65
Alum .................... 29, 67, 90, 150
Ambra ....................27, 29, 127, 151, 289
American Institute ....................32, 350
Amm. car ....................27, 29, 64, 328
Amm. mur ....................27, 52
Anacard. ....................26, 101, 324
Anamnesis of Sycosis. C. von Bœnninghausen .................... 321
Ant. cr. ....................52, 325
Ant. tart. ....................26, 328
Aphorisms on the Affinities of Matter in its Condensation and Attenuation. O. Buchmann. .................... 112
Apis ....................26, 90, 125, 217, 324
Appendix, Cough Repertory ....................45-200
Diarrhœa. P. P. Wells ....................29-47
Dysentery. P. P. Wells ....................1-29
Typhoid Fever. P. P. Wells ...48-87
Arg. met. .................... 26
Arg. nit. ....................29, 125
Arnica ....................26, 27, 186, 206, 326, 356
Arsenic ....................26, 27, 65, 249, 326
Asarum .................... 66
Aurum .................... 67
Australia, Letter from. A. F. Deek .................... 232

Badiaga .................... 352
Baldwin, H. D. Hamamelis in Hæmorrhoids .................... 39
Baptisia .................... 89, 90
Bar. c. ....................27, 105, 150, 328
Belladonna, 26, 38, 64, 96, 132, 135, 247, 324, 289, 305
Benz. ac ....................89, 199
Berberis ....................66, 67, 70
Berridge, E. W. Clinical Cases ....................99, 194
Definitions .................... 53
Case of Gonorrhœa with Some Nuts for the Isopaths to Crack .................... 35
Kali iod. in Phthisis .................... 306
Lac can .................... 218
Ulceration of Cornea Cured by Lac felinum .................... 341
Biegler, J. A. A Clinical Case .................... 193
Book Notices and Reviews. Annual Address before N. Y. Med. Chir. Society. E. P. Fowler .................... 170
Address before California State Hom. Society. J. M. Selfridge .................... 251
American Hom. Pharmacopœia, 2d Edition. J. T. O'Connor. .................... 311
Birth Time in Each Twenty-four Hours. M. M. Walker .................... 40
Burnett's Essays. J. C. Burnett .................... 40
Diseases of Children. B. F. Underwood .................... 138
Gelsemium, Hughes Club .................... 251
Gynæcological Experiences. G. M. Pease .................... 140
Hahnemann, the Founder of Scientific Therapeutics. R. E. Dudgeon .................... 138
Hering's Domestic Physician. C. R. Norton .................... 169
Homœopathy and Gynæcology. Thomas Skinner .................... 251
Homœopathic Treatment of Constipation. H. Bernard .................... 107
The Law of the Similars. C. Wesselhœft .................... 40
Otis Clapp & Son's Visiting List, 40
Plain Talks on Avoided Subjects. H. N. Guernsey .................... 40
Proceedings of the Hom. Society of Ohio .................... 139
Repertory, The Symptoms of Intermittent Fever. W. A. Allen .................... 169
A Simple Solution of the Mystery of Tubercle. R. R. Gregg .................... 140
Supra-pubic Lithotomy. Wm. Tod. Helmuth .................... 107
The Law of Similars .................... 381
Transactions of the Hom. Med. Society of New York .................... 311
Borax ....................27, 90, 325
Bromium ....................66. 89, 90. 101, 198
Bryonia ....................26, 27, 66, 127, 186, 193, 330
Buckmann, O. Aphorisms, etc .................... 112
Pathogenetic Action of High Potencies .................... 257
Bufo ....................67, 89
Butler, C. W. Two Clinical Symptoms .................... 164

Cactus gr .................... 65
Calad .................... 27
Calcarea. ....26, 27, 64, 72, 127, 227, 249, 325
Camph .................... 127
Cann. ind ....................89, 90
Cann. sat .................... 27
Canned Provisions .................... 376
Canth ....................65, 247, 354
Caps .................... 26
Carb. ac. .................... 67
Carbo an ....................26, 64, 90
Carbo veg.. ....................64, 101, 325
Castor ....................66, 67

PAGE
Cauloph. .............................................. 25
Caust.....................26, 27, 64, 90. 127, 216, 324
Central N. Y. Hom. Society, Meetings of..........................85, 179, 223, 253, 315
Central N. Y. Hom. Society, Protest against Eclecticism............................ 378
Cerebro-Nervous Disorders peculiar to Women. Thos Madden Moore, M.D., 372
Cham..........................................26, 27, 64, 221
Chelid...............................................65, 90
China..............................................26, 68, 325
Chloral Hydr. in Lethargic Somnolency. J. C. Burnett.......................... 130
Cicuta.................................................... 65
Cina........................................................ 89
Cinnab........................................................66, 90
Cinchona off., Proving of. B. Fincke.... 44
Clausen, D. W. Address before Central N. Y. Hom. Society.......................... 179
Clinical Cases. E. W. Berridge, 35, 99, 134, 341
J. A. Beigler .................................. 193
C. W. Butler...................................... 164
E. Carlton........................................ 351
Dr. Hannes ...................................... 219
W. A. Hawley .................................... 95
C. F. Millspaugh................................ 280
J. C. Robert....................................... 244
E. Rushmore....................................... 70
Julius Schmitt.................102, 131, 355
S. Seward ........................................ 210
C. L. Swift........................................ 353
J. W. Thomson...............38, 106, 198
Clinical Reflections. Ad. Lippe, 68, 95, 185, 233
Cocculus.........................................64, 90, 326
Coffee....................................................... 65
Colchicum.......................................26 27, 127
Coloc...........................................26, 27, 64, 328
Conium..................................................64, 203
Conscience and Consistency. E. P. Hussy .............................................. 240
Consumption, Contagiousness of, as to the Married ................................ 118
Contagion and Infection. C. T. Harris, 338
Copaiba...................................................66, 216
Correspondence of N. Y. *Med. Times.* P. P. Wells........................................ 74
Creosot.....................................................64, 89
Crocus......................................................... 64
Cross, J. E., Obituary Notice of............ 34
Cuprum.................................................25, 26, 66
Curare ......................................................... 66
Cyclamen.......................................26, 27, 64, 216

Deafness from Accident. R. I. Cooper, 206
Deck, A. F. Letter from Australia ....... 232
Definitions. E. W. Berridge.................. 61
Diarrhœa, Some Peculiar Symptoms of 129
Digitalis.............................................151, 215, 324
Diphtheria and its Hom. Treatment. Ad. Lippe................................................ 120
Distorting Nature.................................. 93
Dr. Lippe and The I. H. A. C. Pearson, 361
Drosera.......................................................... 27
Dulcamara.............................................127, 329
Dunham, Carroll. Seek the Simillimum ..................................................... 1
Dysentery. Merc. corr. in. C. Dunham 53
Dysentery, Lilium Tigrinum. G. M. Pease .................................................... 380

Editorials.
Consultations........................................ 202
Fallacious Theories............................... 313
The I. H. A............................................ 41
The I. H. A. and Its Work.................. 359
Lost Opportunities................................ 283
Micro-Organisms .................................. 109
A New Homœopathy............................ 73
The Renegade Praises the Mongrel................................................. 172
Tattling Doctors.................................... 201
Truth in a Nutshell............................... 202
The War on the Hudson...................... 171
Volume Fourth...................................... 369

Ferrum............................................64, 325, 334
Ferrum mur............................................... 335
Ferrum oxyd.............................................. 221
Festina Lente. W. J. Guernsey............ 346
Fincke, B. Cinch. off............................. 44
Pathogenetic Action of High Potencies.............................................. 257
Potentization......................................... 368
Vaccininum .......................................... 155
Fluor. ac...................................................27, 64

Gamb.......................................................... 89
Gee, W. S. Hours of Aggr. ................ 239
Gelsem.............................................89, 90, 167
Glonoin...................................................89, 90
Gonorrhœa, a Case of, with Some Nuts for the Isopaths to Crack. E. W. Berridge,................................................ 35
Graphites.......................................27, 66, 325
Gratiola ..................................................... 89
Guernsey, W. J. Festina Lente ......... 346
Sebacious Cysts—Conium .......... 203

Hahnemann, the founder of Isopathy. S. Swan................................................. 377
Hahnemann not the founder of Isopathy. E. J. Lee.................................. 378
*Hahnemannian Monthly* and The I. H. A. C. Pearson ............................ 241
Hamamelis.................................................. 89
Harris, C. T. Contagion and Infection..................................................... 338
Hawley, W. A. Retention of Urine, with Hæmaturia.................................. 95
Hepar .............................................26, 27, 326
High Potencies, Pathogenetic Action of. B. Fincke...................................... 257
Homœopathy in Spain. El Palvarez... 230
Hours of Aggr. W. S. Gee.................. 239
Hussey, E. P. Conscience and Consistency..................................................... 240
Hydrast. ..................................................... 89
Hydroph..............................................134, 196
Hyos....................................................26, 64, 89, 90

Ignatia ........................................27, 65, 151, 250
Importance of a Single Symptom. Ad. Lippe..................................................... 149
Indigo ..................................................66, 127
Infallible Diagnosis ................................. 55
I. H. A., Third Annual Meeting......... 255
Editorial ................................................ 41
I. H. A. and its Work, Editorial.......... 359
Iodium............................................27, 326, 351
Ipecac ..........................................................27, 89
Iris v ..........................................................89, 90

Kali b.....................................29, 86, 96, 127, 186
Kali c................26, 27, 64, 89, 134, 151, 325
Kali iod.....................................................65, 304
In Phthisis. E. W. Berridge...... 304
Kalmia ......................................................89, 90
Kobalt.......................................................... 89

Lac. can.....................................................64, 218
Lac felinum.......................160, 194, 195, 341
Lachesis......26, 27, 66, 89, 90, 96, 134, 288, 310, 324, 326
A Lachesis Case. S. Seward........ 310

PAGE

Lachnan....66, 90
Lauro....90, 101, 127, 151
Ledum.... 328
Lept.... 90
Lilium.... 378
Lippe, Ad.
Clinical Reflections....68, 95, 185, 233, 333
Diphtheria and its Homœopathic Treatment.... 120
Importance of a Single Symptom 149
New Discoveries.... 12
Prostate Gland, Diseases of.... 209
Repetition of the Dose.... 364
Revision of the Materia Medica.. 296
Ruta graveolens.... 302
Lobel.... 151
Lyc....26, 27, 64, 90, 99, 150, 216, 221, 325

Mag. c....27, 64, 101
Mag. m....27, 64
Mag. s.... 66
Manc.... 65
McNeil, A. Psoric Theory....293, 349
*Medical Counselor* and I. H. A.... 57
Mental Symptoms of Catamenia.... 65
Merc....26, 64, 127, 135, 325, 333
Merc. corr. in Dysentery. Carroll Dunham.... 53
Mezer.... 325
Mosch....27, 65
Mur. ac.... 29, 328

Natr. c....27, 325
Natr. m....27, 64, 84, 85, 90, 325
New Code.... 94
New Journals.... 382
Notes on Genito-Urinary Therapeutics, 27, 52, 125
Notes and Notices....71, 108, 140, 170, 199, 222, 352, 282, 311, 358, 382
Nitr. ac....57, 64, 128, 324, 326
Nitrum....26, 101
Nux m....27, 64, 219, 220
Nux v....26, 27, 64, 106, 132, 247, 307, 326

Observation Necessary in a Physician. Hahnemann.... 299
Oleand.... 151
Overtaxed Brains. R. R. Gregg.... 18

Pæonia.... 128
Pains in Chest and Back.... 127
Pallad.... 67
Paris.... 27
Pearson, C. *Hahnemannian Monthly* and the I. H. A.... 241
Present and Future of Homœopathy.... 269
Pessaries: Their Use and Abuse. J. Matthews Duncan.... 189
Petrol....66, 151
Petros.... 90
Phelland.... 128
Phosp....26, 27, 64, 103, 128, 325
Ph. ac....27, 66, 326
Pic. acid.... 90
Platinum....26, 64, 132, 324
Plumbum....26, 66, 328
Prostate Gland, Diseases of.... 209
Psoric Theory. A. McNeil....293, 349
Pulsat....26, 27, 64, 95, 213, 219, 325

Quinia, Effects of—Eye and Ear.... 124

Raph.... 128
Remedy and Its Dose, The.... 237

PAGE

Reprints.... 43
Rest as a Cause of Aggravation. C. von Bœnninghausen.... 26
Revision of the Materia Medica. Ad. Lippe.... 296
Rheum....26, 27
Rhodo.... 65
Rhus r.... 129
Rhus t....26, 27, 66, 234, 248, 324
Robert, J. C. A Case of Epileptic Mania 244
Routine Practice. P. P. Wells.... 285
Rushmore, E. Two Berberis Cases.... 70
Ruta....27, 302

Sabad....27, 328
Sabina.... 326
Samb.... 330
Sarsap....90, 354
Sayre and Jacobi, Doctors, on Homœopathy and Homœopathists.... 81
Schmitt, Julius. Cases from My Notebook....102, 131
Sebaceus Cysts—Conium. W. J. Guernsey.... 203
Secale c....27, 65, 327
Seek the Simillimum. C. Dunham.... 1
Selenium....27, 64, 216, 325
Senega.... 26
Sepia....26, 27, 65, 151, 324, 328, 335, 354, 355
Silicea....26, 27, 64, 90, 129, 133, 198, 231
Solis Ictus. J. W. Thomson.... 38
Spigelia....26, 129, 327
Spongia....26, 27
Stannum....26, 27, 64
Staph....26, 27, 325
Stram.... 66, 324
Sulphur 26, 27, 39, 64, 104, 129, 132, 249, 288, 334, 357
Sul. ac.... 330
Swan, S., Clinical Notes.... 357
Lac felinum.... 160
Swift, C. S., Acute Cystitis.... 353
Symph.... 250
Syphilinum.... 194

Tait's Operation, etc.... 91
Tarent.... 64
Thuja....26, 27, 29, 213, 324, 325
Teucrium.... 26
Tobacco Poisoning. J. W. Thomson.... 106
Typhoid Fever, The First Prescription. P. P. Wells.... 2

Urine, Retention of. W. A. Hawley.... 95
Ustilago.... 25

Vaccininum.... 155
Valer.... 26
Verat....90, 151
Vespa.... 64
Viola tr.... 27

Wells, P. P. Addresses to Associated Bodies.... 141
Appendix. Diarrhœa, Dysentery, Typhoid Fever.... 1-87
Correspondence of N. Y. *Medical Times*.... 74
Hahnemann's Chronic Miasms... 175
Routine Practice.... 285
Typhoid Fever, The First Prescription.... 2

Xanthox.... 25

Zincum....65, 129, 327
Zing.... 66

Vol. III. DECEMBER, 1883. No. 12.

### THE HOMŒOPATHIC PHYSICIAN,

A MONTHLY JOURNAL OF MEDICAL SCIENCE.

*"Magna est veritas, et prævalebit."*

E. J. LEE, M. D.,
EDITOR.

CONTENTS:

(See inside of Cover.)

NOTICE.

***All articles for publication, books for review, business communications, etc., must be sent to Editor, 2109 Chestnut Street, Philadelphia.***

